# Ethyl (*E*)-2-(2-furyl­idene)hydrazine­carboxyl­ate

**DOI:** 10.1107/S1600536809023654

**Published:** 2009-06-24

**Authors:** Lu-Ping Lv, Wei-Wei Li, Tie-Ming Yu, Wen-Bo Yu, Xian-Chao Hu

**Affiliations:** a Department of Chemical Engineering, Hangzhou Vocational and Technical College, Hangzhou 310018, People’s Republic of China; b Research Center of Analysis and Measurement, Zhejiang University of Technology, Hangzhou 310014, People’s Republic of China

## Abstract

In the title compound, C_8_H_10_N_2_O_3_, the hydrazinecarboxyl­ate group is twisted from the furan ring by 6.98 (17)°. In the crystal, the mol­ecules are linked into one-dimensional chains running along the *c* axis by N—H⋯O hydrogen bonds.

## Related literature

For general background, see: Parashar *et al.* (1988 ▶); Hadjoudis *et al.* (1987 ▶); Borg *et al.* (1999 ▶); Kahwa *et al.* (1986 ▶); Santos *et al.* (2001 ▶). For a related structure, see: Shang *et al.* (2007 ▶).
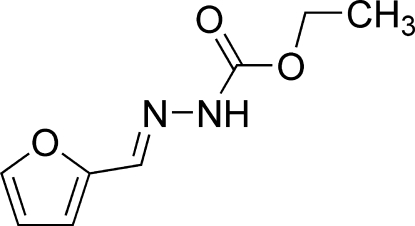


## Experimental

### 

#### Crystal data

C_8_H_10_N_2_O_3_
*M* *_r_* = 182.18
Monoclinic, 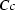

*a* = 14.150 (6) Å
*b* = 9.285 (5) Å
*c* = 8.108 (4) Å
β = 118.540 (16)°
*V* = 935.8 (8) Å^3^
*Z* = 4
Mo *K*α radiation
μ = 0.10 mm^−1^
*T* = 223 K
0.24 × 0.22 × 0.17 mm

#### Data collection

Bruker SMART CCD area-detector diffractometer
Absorption correction: multi-scan (*SADABS*; Bruker, 2002 ▶) *T*_min_ = 0.975, *T*_max_ = 0.985
2344 measured reflections
816 independent reflections
733 reflections with *I* > 2σ(*I*)
*R*_int_ = 0.024

#### Refinement

*R*[*F* ^2^ > 2σ(*F* ^2^)] = 0.028
*wR*(*F* ^2^) = 0.071
*S* = 1.07
816 reflections
118 parameters
H-atom parameters constrained
Δρ_max_ = 0.10 e Å^−3^
Δρ_min_ = −0.16 e Å^−3^

### 

Data collection: *SMART* (Bruker, 2002 ▶); cell refinement: *SAINT* (Bruker, 2002 ▶); data reduction: *SAINT*; program(s) used to solve structure: *SHELXS97* (Sheldrick, 2008 ▶); program(s) used to refine structure: *SHELXL97* (Sheldrick, 2008 ▶); molecular graphics: *SHELXTL* (Sheldrick, 2008 ▶); software used to prepare material for publication: *SHELXTL*.

## Supplementary Material

Crystal structure: contains datablocks I, global. DOI: 10.1107/S1600536809023654/bg2272sup1.cif

Structure factors: contains datablocks I. DOI: 10.1107/S1600536809023654/bg2272Isup2.hkl

Additional supplementary materials:  crystallographic information; 3D view; checkCIF report

## Figures and Tables

**Table 1:** Hydrogen-bond geometry (Å, °)

*D*—H⋯*A*	*D*—H	H⋯*A*	*D*⋯*A*	*D*—H⋯*A*
N2—H2⋯O2^i^	0.86	2.08	2.916 (3)	164

Symmetry code: (i) 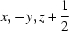.

**Fig. 1.**
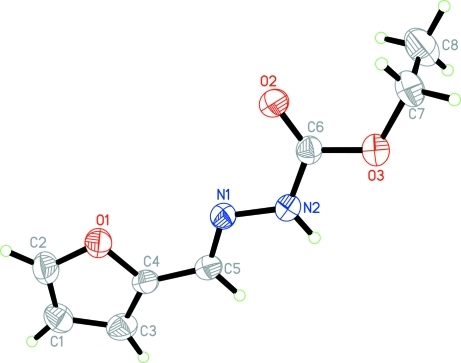
The asymmetric unit of (I). Displacement ellipsoids are drawn at the 40% probability level.

**Fig. 2.**
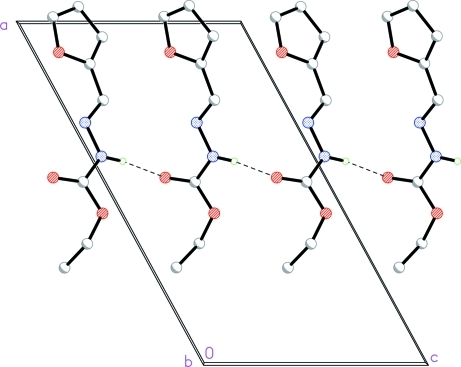
Crystal packing of (I), showing the formation of chains along c. Hydrogen bonds are shown as dashed lines.

## supplementary crystallographic information

### Comment

Benzaldehydehydrazone derivatives have attracted much attention due to their pharmacological activity (Parashar *et al.*, 1988) and their photochromic properties (Hadjoudis *et al.*, 1987). They are important intermidiates of 1,3,4-oxadiazoles, which have been reported to be versatile compounds with many interesting properties (Borg *et al.*, 1999). Metal complexes based on Schiff bases have received considerable attention because they can be utilized as model compounds of active centres in various proteins and enzymes (Kahwa *et al.*, 1986; Santos *et al.*, 2001). We report here the crystal structure of the title compound (Fig. 1).

In the title compound, C_8_H_10_N_2_O_3_ (I), the N1/N2/O2/O3/C6/C7 planes form dihedral angles of 6.98 (17)° with the O1/C1—C4 planes.The bond lengths and angles are comparable to those observed for methyl*N*'-[(*E*)-4-methoxybenzylidene]hydrazinecarboxylate (Shang *et al.*, 2007).

In the crystal structure, the molecules are linked into one-dimentional chains running along the *c* axis by N—H···O hydrogen bonds(Table 1,Fig.1).

### Experimental

Furfuraldehyde (0.96 g, 0.01 mol) and Ethyl hydrazinecarboxylate (1.04 g, 0.01 mol) were dissolved in stirred methanol (20 ml) and left for 3 h at room temperature. The resulting solid was filtered off and recrystallized from ethanol to give the title compound in 95% yield. Single crystals suitable for X-ray analysis were obtained by slow evaporation of an ethanol solution at room temperature (m.p. 410–412 K).

### Refinement

H atoms were positioned geometrically (N—H = 0.86 Å and C—H = 0.93 or 0.96 Å) and refined using a riding model, with *U*_iso_(H) = 1.2*U*_eq_(C,*N*) and 1.5*U*_eq_(C_methyl_). In the absence of significant anomalous scattering effects, Friedel pairs were averaged.

### Crystal data

**Table:**

C_8_H_10_N_2_O_3_	*F*(000) = 384
*M**_r_* = 182.18	*D*_x_ = 1.293 Mg m^−^^3^
Monoclinic, *C**c*	Mo *K*α radiation, λ = 0.71073 Å
Hall symbol: C -2yc	Cell parameters from 1451 reflections
*a* = 14.150 (6) Å	θ = 2.7–25.0°
*b* = 9.285 (5) Å	µ = 0.10 mm^−^^1^
*c* = 8.108 (4) Å	*T* = 223 K
β = 118.540 (16)°	Block, colourless
*V* = 935.8 (8) Å^3^	0.24 × 0.22 × 0.17 mm
*Z* = 4	

### Data collection

**Table:**

Bruker SMART CCD area-detector diffractometer	816 independent reflections
Radiation source: fine-focus sealed tube	733 reflections with *I* > 2σ(*I*)
graphite	*R*_int_ = 0.024
φ and ω scans	θ_max_ = 25.0°, θ_min_ = 2.7°
Absorption correction: multi-scan (*SADABS*; Bruker, 2002)	*h* = −16→16
*T*_min_ = 0.975, *T*_max_ = 0.985	*k* = −10→10
2344 measured reflections	*l* = −8→9

### Refinement

**Table:**

Refinement on *F*^2^	Primary atom site location: structure-invariant direct methods
Least-squares matrix: full	Secondary atom site location: difference Fourier map
*R*[*F*^2^ > 2σ(*F*^2^)] = 0.028	Hydrogen site location: inferred from neighbouring sites
*wR*(*F*^2^) = 0.071	H-atom parameters constrained
*S* = 1.07	*w* = 1/[σ^2^(*F*_o_^2^) + (0.0384*P*)^2^ + 0.0727*P*] where *P* = (*F*_o_^2^ + 2*F*_c_^2^)/3
816 reflections	(Δ/σ)_max_ < 0.001
118 parameters	Δρ_max_ = 0.10 e Å^−^^3^
0 restraints	Δρ_min_ = −0.16 e Å^−^^3^

### Special details

**Table:**

Geometry. All e.s.d.'s (except the e.s.d. in the dihedral angle between two l.s. planes) are estimated using the full covariance matrix. The cell e.s.d.'s are taken into account individually in the estimation of e.s.d.'s in distances, angles and torsion angles; correlations between e.s.d.'s in cell parameters are only used when they are defined by crystal symmetry. An approximate (isotropic) treatment of cell e.s.d.'s is used for estimating e.s.d.'s involving l.s. planes.
Refinement. Refinement of *F*^2^ against ALL reflections. The weighted *R*-factor *wR* and goodness of fit *S* are based on *F*^2^, conventional *R*-factors *R* are based on *F*, with *F* set to zero for negative *F*^2^. The threshold expression of *F*^2^ > σ(*F*^2^) is used only for calculating *R*-factors(gt) *etc*. and is not relevant to the choice of reflections for refinement. *R*-factors based on *F*^2^ are statistically about twice as large as those based on *F*, and *R*- factors based on ALL data will be even larger.

### Fractional atomic coordinates and isotropic or equivalent isotropic displacement parameters (Å^2^)

**Table:**

	*x*	*y*	*z*	*U*_iso_*/*U*_eq_	
O3	−0.05924 (14)	−0.0975 (2)	0.4141 (2)	0.0686 (5)	
N2	0.10734 (15)	−0.0174 (2)	0.5472 (3)	0.0561 (5)	
H2	0.0934	0.0188	0.6309	0.067*	
O1	0.41276 (15)	0.0404 (2)	0.6109 (3)	0.0754 (6)	
O2	0.04565 (14)	−0.1545 (2)	0.2815 (2)	0.0654 (5)	
N1	0.20660 (15)	0.0030 (2)	0.5573 (3)	0.0527 (5)	
C3	0.4583 (2)	0.1980 (3)	0.8408 (4)	0.0702 (8)	
H3	0.4555	0.2580	0.9302	0.084*	
C5	0.27221 (19)	0.0837 (3)	0.6911 (3)	0.0541 (6)	
H5	0.2503	0.1248	0.7719	0.065*	
C6	0.03274 (18)	−0.0952 (3)	0.4038 (3)	0.0531 (6)	
C4	0.37893 (19)	0.1127 (2)	0.7200 (3)	0.0533 (6)	
C7	−0.1479 (2)	−0.1819 (4)	0.2726 (4)	0.0785 (8)	
H7A	−0.1895	−0.2234	0.3273	0.094*	
H7B	−0.1196	−0.2601	0.2299	0.094*	
C1	0.5472 (2)	0.1804 (3)	0.8079 (4)	0.0745 (8)	
H1	0.6138	0.2259	0.8705	0.089*	
C2	0.5161 (2)	0.0859 (4)	0.6694 (5)	0.0822 (9)	
H2A	0.5589	0.0544	0.6183	0.099*	
C8	−0.2181 (3)	−0.0897 (5)	0.1115 (5)	0.1058 (12)	
H8A	−0.2758	−0.1465	0.0190	0.159*	
H8B	−0.1769	−0.0492	0.0571	0.159*	
H8C	−0.2473	−0.0135	0.1537	0.159*	

### Atomic displacement parameters (Å^2^)

**Table:**

	*U*^11^	*U*^22^	*U*^33^	*U*^12^	*U*^13^	*U*^23^
O3	0.0582 (10)	0.0793 (13)	0.0731 (12)	−0.0042 (9)	0.0354 (9)	−0.0022 (10)
N2	0.0546 (12)	0.0655 (13)	0.0533 (11)	0.0001 (10)	0.0299 (9)	−0.0041 (10)
O1	0.0585 (10)	0.0904 (12)	0.0779 (12)	−0.0088 (10)	0.0329 (10)	−0.0277 (11)
O2	0.0669 (10)	0.0755 (11)	0.0593 (10)	−0.0093 (9)	0.0347 (9)	−0.0106 (9)
N1	0.0517 (11)	0.0574 (11)	0.0510 (11)	0.0013 (9)	0.0261 (9)	0.0025 (9)
C3	0.081 (2)	0.0659 (17)	0.0638 (16)	−0.0113 (15)	0.0347 (15)	−0.0160 (13)
C5	0.0610 (14)	0.0538 (14)	0.0474 (13)	0.0032 (13)	0.0259 (11)	0.0004 (12)
C6	0.0517 (13)	0.0586 (15)	0.0521 (14)	0.0045 (11)	0.0273 (11)	0.0110 (12)
C4	0.0594 (14)	0.0537 (13)	0.0451 (12)	0.0034 (11)	0.0236 (11)	−0.0002 (11)
C7	0.0616 (16)	0.082 (2)	0.092 (2)	−0.0168 (16)	0.0366 (16)	−0.0043 (17)
C1	0.0593 (14)	0.0745 (19)	0.0787 (19)	−0.0163 (14)	0.0241 (14)	−0.0068 (15)
C2	0.0567 (15)	0.099 (2)	0.095 (2)	−0.0099 (15)	0.0392 (15)	−0.0218 (19)
C8	0.0683 (19)	0.126 (3)	0.103 (3)	−0.012 (2)	0.0241 (19)	0.006 (2)

### Geometric parameters (Å, °)

**Table:**

O3—C6	1.344 (3)	C5—C4	1.438 (4)
O3—C7	1.459 (3)	C5—H5	0.9300
N2—C6	1.347 (3)	C7—C8	1.479 (5)
N2—N1	1.380 (2)	C7—H7A	0.9700
N2—H2	0.8600	C7—H7B	0.9700
O1—C2	1.371 (4)	C1—C2	1.324 (4)
O1—C4	1.366 (3)	C1—H1	0.9300
O2—C6	1.221 (3)	C2—H2A	0.9300
N1—C5	1.279 (3)	C8—H8A	0.9600
C3—C4	1.341 (4)	C8—H8B	0.9600
C3—C1	1.415 (4)	C8—H8C	0.9600
C3—H3	0.9300		
			
C6—O3—C7	117.0 (2)	O3—C7—C8	110.2 (3)
C6—N2—N1	118.71 (18)	O3—C7—H7A	109.6
C6—N2—H2	120.6	C8—C7—H7A	109.6
N1—N2—H2	120.6	O3—C7—H7B	109.6
C2—O1—C4	105.7 (2)	C8—C7—H7B	109.6
C5—N1—N2	115.90 (18)	H7A—C7—H7B	108.1
C4—C3—C1	107.7 (2)	C2—C1—C3	105.7 (2)
C4—C3—H3	126.1	C2—C1—H1	127.1
C1—C3—H3	126.1	C3—C1—H1	127.1
N1—C5—C4	121.9 (2)	C1—C2—O1	111.5 (3)
N1—C5—H5	119.1	C1—C2—H2A	124.3
C4—C5—H5	119.1	O1—C2—H2A	124.3
O2—C6—O3	124.6 (2)	C7—C8—H8A	109.5
O2—C6—N2	125.6 (2)	C7—C8—H8B	109.5
O3—C6—N2	109.7 (2)	H8A—C8—H8B	109.5
C3—C4—O1	109.4 (2)	C7—C8—H8C	109.5
C3—C4—C5	132.7 (2)	H8A—C8—H8C	109.5
O1—C4—C5	117.9 (2)	H8B—C8—H8C	109.5

### Hydrogen-bond geometry (Å, °)

**Table:**

*D*—H···*A*	*D*—H	H···*A*	*D*···*A*	*D*—H···*A*
N2—H2···O2^i^	0.86	2.08	2.916 (3)	164

Symmetry codes: (i) *x*, −*y*, *z*+1/2.
